# Integrating Large Language Models Into Patient Health Education: Interview Study Among Nurses

**DOI:** 10.2196/102846

**Published:** 2026-11-12

**Authors:** Xiaoqing Tang, Qianqian Zhang, Sulin Xu, Qiyang Hong, Zhongfan Kan, Yiming Gao, Yuebing Du, Yujie Zhao, Dan Xu, Guiying Liu, Huijuan Zhang, Jinying Zhao, Zhen Song, Peixing Wan, Erlie Jiang, Wenjun Xie

**Affiliations:** 1 Tianjin University of Traditional Chinese Medicine, Tianjin, China; 2 Institute of Hematology and Blood Diseases Hospital, No. 288, Nanjing Road, Tianjin, China; 3 State Key Laboratory of Respiratory Health and Multimorbidity, Institute of Basic Medical Sciences, Chinese Academy of Medical Sciences and Peking Union Medical College, Beijing, China; 4 Department of Medical Bioinformatics, School of Basic Medical Sciences, Peking University, Beijing, China

**Keywords:** generative artificial intelligence, health education, large language models, nursing, qualitative research

## Abstract

**Background:**

Large language models (LLMs) have shown potential to support patient education, improve information accessibility, and assist with repetitive clinical communication tasks. However, the clinical integration of LLMs depends not only on technological capabilities but also on how these tools fit within existing health care workflows. Existing studies have largely focused on technical performance of LLMs or the experiences of physicians and patients, and little is known about how frontline nurses, who play a central role in patient education, experience LLM-supported education workflows in clinical practice settings.

**Objective:**

This study aimed to explore frontline nurses’ experiences with an LLM-assisted patient education workflow, including perceived benefits, changes, challenges, and considerations for clinical practice.

**Methods:**

Semistructured interviews were conducted with 16 frontline hematological nurses between December 2025 and March 2026 at the Institute of Hematology and Blood Diseases Hospital, Chinese Academy of Medical Sciences to explore their experiences. Data were analyzed using an inductive codebook thematic analysis, with codes and themes iteratively reviewed and refined through researcher discussions.

**Results:**

Nurses’ experiences were characterized by 3 interconnected tensions. First, nurses perceived a balance between efficiency gains and information safety concerns, as the LLM-assisted workflow supported more efficient and standardized information delivery while requiring additional oversight. Second, nurses perceived changes in their roles and responsibilities, accompanied by increased demands for professional knowledge and competencies. Third, nurses perceived potential patient benefits of LLM use particularly in facilitating access to specialized health information, while identifying barriers related to limited health literacy and limited familiarity with LLM-based tools.

**Conclusions:**

From nurses’ perspectives, LLM use was perceived to support more efficient delivery of patient health information while also requiring attention to information safety, professional responsibility, and patient-related challenges. These findings highlight that nurses’ roles remain important in supporting the safe and appropriate use of LLM-assisted education. Future research and implementation efforts should explore workflow-oriented design strategies for integrating LLMs into clinical nursing practice while considering nurse oversight, support clinical workflow adaptation, and address patient needs to support safe and appropriate use.

## Introduction

Large language models (LLMs) have rapidly emerged as important applications of AI in health care. Owing to their strong natural language processing capabilities, LLMs have demonstrated considerable potential—including analyzing clinical information [1], supporting medical decision-making [2], generating patient education materials, and responding to patient-specific questions through conversational interactions [3]—to revolutionize health care delivery [2]. Recent studies have demonstrated LLMs’ potential in clinical settings, including their application in patient health education. By assessing educational materials on pediatric cataracts generated by ChatGPT, Dihan et al [4] showed how LLMs could enhance conventional clinical health education. A cross-sectional study of 200 ophthalmology questions found LLM answers comparable to physician answers in safety and error rates [5].

Therapeutic patient education aims to help patients more effectively understand and manage their own conditions [6], which directly affects patients’ self-management and subsequent quality of life [7]. In hematology, this need is particularly acute. Patients who often undergo unique, complex treatments such as hematopoietic cell transplantation and chimeric antigen receptor T-cell therapy have high information needs [8], and clinical health education occupies a substantial portion of nurses’ daily work. At the same time, health care professionals continue to face challenges such as heavy workloads, time-pressured decision-making, and the management of large volumes of heterogeneous clinical information [9]. Traditional approaches often struggle to address these challenges efficiently. In this context, integrating the information-processing capabilities of LLMs into clinical workflows has become an important area of interest.

The clinical application of LLMs requires not only technological capabilities but also appropriate integration into existing health care workflows. However, existing studies have primarily focused on the technical performance of LLMs or the experiences of physicians or patients [10,11], while less is known about how nurses experience LLM use in patient education, particularly in complex hematology settings. As frontline clinicians closely involved in patient education, nurses play a central role in translating health information into patient-centered communication. Although quantitative studies provide important evidence regarding LLM accuracy, performance, and adoption, they may not fully capture how clinical users experience and adapt to these technologies in real-world workflows [12]. Therefore, this study aimed to explore frontline nurses’ experiences and perceptions of LLM-assisted patient education, with a focus on perceived changes, challenges, and safety considerations in clinical practice.

## Methods

### Study Design

This exploratory qualitative study aimed to explore nurses’ experiences with an LLM-assisted health education workflow in clinical practice. Interviews were conducted after completion of the intervention phase and all outcome data collection of the parent randomized controlled trial (RCT) which investigated an LLM-assisted health education intervention compared with traditional nurse-led education among patients with hematological diseases. During the 6-month intervention period, nurses gained experience implementing the LLM-assisted health education workflow in clinical practice.

All interviewed nurses were recruited from the intervention team involved in the LLM-assisted health education workflow evaluated in this RCT. Patients interacted directly with LLM during the workflow, while nurses supported the process by introducing LLM, providing instructions before use, and reviewing patient-LLM interactions after completion as part of clinical workflow. During this review process, nurses could identify perceived information gaps or safety concerns and provided supplementary education or clarification when needed. These nurses did not participate in real-time patient-LLM interactions. The present study analyzed only nurses’ interview data and did not examine the effectiveness outcomes of the parent trial.

### Study Participants

Nurses involved in the LLM-assisted health education workflow evaluated in the parent RCT were invited to participate in the qualitative study. A purposive sampling approach was used, with all eligible nurses who had direct experience with the LLM-supported health education workflow invited to participate. This enabled them to provide firsthand accounts of their experiences, perceived benefits, challenges, and safety considerations. All eligible nurses were invited and all agreed to participate in the interviews.

Sample adequacy was assessed based on the concept of information power proposed by Malterud et al [13]. Information power was considered throughout the recruitment and analysis process. The study had a focused aim of exploring nurses’ experiences with the LLM-supported health education workflow. Participants had high sample specificity because all were frontline nurses with direct experience of the LLM-supported health education workflow. Semistructured interviews provided rich and detailed accounts of participants’ experiences. The adequacy of the sample was further supported by the richness of the interview data and the consistency of identified patterns during analysis. Considering these factors, the final sample of 16 participants was considered adequate to address the research question.

### Technical Platforms and Research Tools

The SSPEC (site-specific prompt engineering chatbot) LLM-assisted patient education system jointly developed and deployed by the Institute of Basic Medical Sciences, Chinese Academy of Medical Sciences, Peking Union Medical College, and School of Basic Medical Sciences, Peking University, serves as the technical platform for this work [14]. The system was designed to provide patient-oriented health education knowledge, using the hfl/chinese-alpaca-2-7b model as the underlying LLM. Detailed information regarding the model is provided in Multimedia Appendix 1.

### Data Collection

Semi-structured, in-depth interviews were conducted at the Institute of Hematology and Blood Diseases Hospital, Chinese Academy of Medical Sciences in Tianjin, China, from December 2025 to March 2026, after completion of the RCT and related data collection. To guarantee uniformity, all interviews were conducted by the same researchers, who received in-depth training in qualitative interviewing and were guided by an expert in qualitative methods, including practice interviews prior to data collection. The interviews began with brief questions about participants’ demographic characteristics and previous experience with LLMs and AI-based tools. Participants were then encouraged to freely discuss their experiences with the LLM-supported health education workflow, including perceived benefits, challenges, changes in clinical practice, and safety considerations, in Chinese. Every interview was recorded while taking place in the hospital’s interview room. The audio interviews lasted between 37 and 53 minutes. Field notes were used to record reflections, body language, and facial emotions. Recordings were transcribed verbatim within 24 hours and double-checked by 2 researchers. The written records were supplemented with pertinent field notes. For reporting purposes, selected quotations were translated into English by the research team.

### Interview Guide

We prepared a semistructured interview guide based on the research objectives to thoroughly examine participants’ experience (detail is provided in Multimedia Appendix 1). To ensure clarity and appropriateness, this interview guide was pretested with a group of clinical nurses and nursing researchers with relevant professional experience who were not part of the main study, and it was modified in response to their opinions.

### Data Analysis

Interview data were analyzed using an inductive codebook thematic analysis approach, following the phases of thematic analysis described by Braun and Clarke [15]. The interview transcripts were originally analyzed in Chinese. Selected quotations were translated into English and verified against the original Chinese transcripts by the research team to ensure semantic accuracy and consistency. No back-translation or formal cross-cultural validation was conducted, as the translations were used solely for reporting participant quotations. An inductive approach was used to explore nurses’ experiences with LLM-assisted health education. Two researchers independently read the transcripts several times and conducted line-by-line coding to identify initial codes from participants’ descriptions of their experiences with the LLM-assisted health education workflow. Coding was conducted inductively without using predefined categories. The researchers compared and discussed the codes during the analysis process and any differences were resolved through discussion. The initial coding framework was iteratively refined as the analysis progressed. Related codes were compared to identify connections in meaning and codes reflecting related aspects of nurses’ experiences were brought together to develop preliminary themes. The researchers reviewed these themes against the coded data and full transcripts to assess whether they adequately supported by the data and were sufficiently distinct from one another. The themes were further refined through team discussion to establish the final thematic structure. This study is reported in accord with the Standards for Reporting Qualitative Research (SRQR) reporting guideline [16]. The completed SRQR checklist is provided in Checklist 1.

### Rigor

This study used the following quality control techniques based on criteria from Lincoln and Guba [17]: (1) confirmability (researchers engaged in reflexive discussions throughout data collection and analysis to consider their assumptions, interpretations, and potential influences on the findings); (2) transferability (detailed presentation of participants’ backgrounds and the clinical context was used to facilitate assessment of the applicability of the findings to other settings); (3) dependability (discussion and resolution of coding discrepancies were conducted, along with iterative refinement of the coding framework throughout the analysis process); and (4) credibility (researcher discussions and review of preliminary themes took place to ensure that the findings accurately reflected the interview data) [18].

### Researcher Reflexivity

The interviewer was involved in coordinating the parent RCT but was not involved in intervention delivery or outcome assessment. The interviewer received training in qualitative interviewing and maintained a reflexive stance throughout data collection and analysis. No supervisory relationship existed between the interviewer and participants, and nurses were encouraged to share both positive and critical experiences.

### Ethics Declarations

This study was approved by the Institute of Hematology and Blood Diseases Hospital, Chinese Academy of Medical Sciences (number: QTJC2025066-EC-1). The parent trial was registered at the Chinese Clinical Trial Registry (registration number: ChiCTR2500107909). The study adhered to the principles of the Declaration of Helsinki [19]. Written informed consent was obtained from all nurse participants before interviews. All interview data were deidentified and participants were assigned unique identification codes during transcription and analysis. Access to the data was limited to the research team. No patient-identifiable information from LLM interactions was included in the qualitative dataset. They were compensated for their participation via leave arrangements and salary support, funded by the research grant. To avoid influencing participation or interview responses, participants were informed of the leave and salary support arrangements only after all interviews had been completed.

### Data Availability

Due to confidentiality and privacy concerns related to interview participants, the original interview transcripts are not publicly available. Additional information regarding the study methods may be available from the corresponding author upon reasonable request.

## Results

### Demographic Characteristics of Participants

The demographic characteristics of the 16 hematology nurses are presented in Table 1, and the themes and subthemes identified from the interviews are presented in Table 2.

**Table 1.** Characteristics of hematology nurses participating in the study (N=16). Note: Due to rounding, percentages may not sum to 100%.

Characteristic	Value
Biological sex, n (%)	
Female	13 (81.3)
Male	3 (18.8)
Age (years), median (IQR)	34 (28.5‐38.5)
Education, n (%)	
Bachelor’s degree	6 (37.5)
Master’s degree	6 (37.5)
Associate degree	4 (25.0)
Professional title, n (%)	
Registered nurse	10 (62.5)
Supervising nurse	3 (18.8)
Associate chief nurse	3 (18.8)
Experience (years), median (IQR)	8.0 (6.0-10.5)
Daily health education time (hours), median (IQR)	2.3 (1.5‐3.0)
Prior experience with LLMs^a^, n (%)	
Had heard of LLMs	2 (12.5)
Had used LLM tools	10 (62.5)
Had experience with AI-based tools at work	4 (25.0)

a LLM: large language model.

**Table 2.** Themes and subthemes identified from interviews with hematology nurses.

Themes	Subthemes
Balancing efficiency gains and safety concerns	Supporting efficiency and standardization of health education Information reliability concerns and perceived oversight burden
Changing nursing roles and professional demands	Perceived changes in nurses’ roles Perceived increase in professional responsibility Perceived competence demands among nurses
Balancing patient empowerment and communication challenges	Patient empowerment through active learning Changes in nurse-patient communication Patient-related barriers to LLM^a^-supported education

a LLM: large language model.

### Theme 1: Balancing Efficiency Gains and Safety Concerns

#### Supporting Efficiency and Standardization of Health Education

Participants observed that the LLM-assisted workflow could support the efficiency and organization of nursing health education by rapidly generating structured educational content, helping nurses cover key educational points, and promoting greater consistency across nurses. One participant noted that “the educational content generated by the LLM was consistent with our institutional standards” (nurse 2), which contributed to greater consistency in health education. Nurses also noticed that patients could “access educational information beyond a single department, thereby expanding content coverage” (nurse 1). Several participants also observed that patients viewed the hospital-based LLM system a more trustworthy and structured source of online information. As one participant explained:

*Compared with online information, our educational content is more standardized and reliable.*

[Nurse 6]

Participants further noticed that LLM use may reduce some repetitive educational tasks and potentially allow nurses to spend more time on other aspects of care. One participant recalled that before LLM use, she often repeated the same educational content “no fewer than three times” per day. After using LLM, nurses spent less time answering repeated questions from patients. As one participant described:

*I no longer get repeatedly stopped by patients asking the same questions, which gives me more time to focus on other nursing tasks.*

[Nurse 12]

Overall, participants noticed that LLM use helped organize health education content, promote consistency in educational delivery, and potentially reduce some repetitive teaching tasks, which may allow nurses to devote more time to other clinical responsibilities.

#### Information Reliability Concerns and Perceived Oversight Burden

Participants described perceived limitations in LLM-generated responses and concerns regarding information safety during clinical use. One participant explained:

*Sometimes the content does not fully match the clinical situation, so I have to revise it and emphasize the correct information to the patient.*

[Nurse 5]

Another participant added:

*If we forget to check afterwards, patients might get potentially inaccurate or inappropriate information and we won’t correct it in time.*

[Nurse 7]

Participants described observed limitations in LLM-generated responses and concerns regarding information safety during clinical use, including missing information requiring supplementation and responses requiring further clarification.

In response to these concerns, nurses described the need for additional review and supervision. One participant stated, “I need to guide the patient to ask questions” (nurse 10), illustrating that nurses needed to provide additional support to facilitate appropriate LLM use, which nurses perceived as requiring additional time demands and effort. Nurses described the need to review interaction records after completion, revise inappropriate responses that did not fully align with clinical contexts, and add missing information, especially for complex or specialized questions. As a result, participants noticed that supporting safe and appropriate information delivery introduced additional review and oversight efforts.

### Theme 2 Changing Nursing Roles and Professional Demands

#### Perceived Changes in Nurses’ Roles

Participants perceived LLM support changes their role in this workflow. Some nurses noted that LLM support could reduce communication-related burden during health education, particularly for nurses who felt less confident in face-to-face communication. However, participants emphasized that LLMs could not replace nurses’ clinical judgment. Instead, they described that nurses continued to play a role in providing clinical oversight by reviewing patient-LLM interaction records after completion, identifying potential information gaps or safety concerns, and offering clarification when needed. A junior nurse with 2 years of clinical experience expressed this concern:

*I feel I need more professional knowledge because I have to judge whether the model’s responses are correct.*

[Nurse 1]

These changes in nurses’ roles were accompanied by increased responsibility for ensuring the quality and safety of health education.

#### Perceived Increase in Professional Responsibility

At the same time, participants emphasized that the responsibility for education quality remained with nurses despite the involvement of LLMs. One nurse explained:

*Using LLMs definitely amplifies professional value because you add this extra step. It’s very helpful for patients, but you also have to learn to evaluate the quality of LLM conversations. Greater responsibility means greater professional value.*

[Nurse 16]

All participants emphasized that quality accountability stays with nurses, with the idea that “quality issues ultimately fall back on nurses because we still hold the decision-making power” (Nurse 11). A nurse with more than 10 years of clinical experience emphasized:

*The risk lies with the nurse, health education is inherently a component of nurses’ professional responsibilities, fully responsible for the entire process and content. So responsibility actually increases.*

[Nurse 11]

The expanded responsibility associated with LLM-assisted education also led nurses to recognize new competency requirements.

#### Perceived Competence Demands Among Nurses

With these expanded roles and responsibilities, participants also perceived new competence demands and related pressure. A nurse with 8 years of experience said:

*When patients use large language models, they tend to have more questions.*

[Nurse 2]

Another participant added:

*Some of their questions can be quite unexpected—you wouldn’t see them coming.*

[Nurse 7]

A junior nurse further stated:

*I can hardly keep up with patients’ questions sometimes. The introduction of LLM puts pressure on me and makes me realize I need to improve myself.*

[Nurse 1]

Thus, while LLM use encouraged nurses to strengthen their clinical knowledge and critical thinking, it also created new competence demands and increased professional pressure, particularly those with less experience.

### Theme 3 Balancing Patient Empowerment and Communication Challenges

#### Patient Empowerment Through Active Learning

Participants noted that LLM use may support patients’ engagement in self-care by encouraging them to actively seek disease-related information. They described a perceived shift from patients being passive recipients of health education toward more actively engaged participants in their own care when engaging with LLM-supported education.

*They gain significant benefits in their subsequent self-care for the disease. Patients must participate in and understand any follow-up treatment and care.*

[Nurse 4]

Participants also observed that patients who are more self-directed or highly educated might be more likely to have a clear understanding of their condition and priorities. One participant said:

*They know their needs and take the initiative, asking the LLM relevant questions, which may be especially beneficial for this group of patients.*

[Nurse 12]

#### Changes in Nurse-Patient Communication

Participants also described how LLM use influenced their perceptions of nurse-patient communication. On the positive side, one participant described reduced interpersonal tension, stating:

*Before, I often provided health education, but some patients still felt that they didn’t get enough information. I remember one patient complained that I hadn’t provided education, and I felt really misunderstood.*

[Nurse 9]

Additionally, nurses gained new insights into patient questions and concerns by reviewing LLM conversations. One participant explained:

*By reviewing the conversations between patients and the LLM, we actually get to know what patients really need. There are questions they would never ask us—like their survival rate or medication issues...I think we can even dig into the questions patients ask the LLM to figure out what really matters to them. Maybe that’s exactly what we need to focus on next in their care.*

[Nurse 15]

However, someone noticed a reduction in face-to-face interaction. One participant pointed out:

*We’re having less face-to-face communication with patients. Some nursing issues can only be identified through direct interaction with patients.*

[Nurse 11]

Despite the capabilities of LLM, participants emphasized aspects of nursing that technology cannot replace. All of the participants agreed, with one stating:

*When we provide health education, we always offer humanistic care based on the patient’s situation, the model cannot fully replicate this aspect of nursing care.*

[Nurse 12]

#### Patient-Related Barriers to LLM-Supported Education

Participants described patient-related factors that could influence engagement with LLM-supported education. First, one nurse explained:

*MDS/MPN patients are mostly older adults, and many of them do not even know what these terms mean.*

[Nurse 12]

Several participants reported that patients sometimes lacked clear questions to ask. One participant explained:

*Patients do not know what they should ask, especially when they are newly admitted and still confused about their condition, while some patients only care about treatment outcomes.*

[Nurse 3]

Participants described challenges with patients who had limited literacy or unfamiliarity with technology. One participant said:

*He thought he couldn’t use it, because he can’t type and doesn’t read well, so he was reluctant to use the tool.*

[Nurse 8]

Nurses also noted that LLM responses could be too academic or technical for some patients, requiring translation or explanation:

*The model’s output is often formal and academic, and some patients may not understand it.*

[Nurse 6]

High-risk or severely ill patients were considered unsuitable for independent LLM use by participants, and nurses emphasized supervision and follow-up. Overall, participants described these limitations placed restrictions on which patients could effectively use the LLM.

## Discussion

### Principal Results

Three key tensions characterized nurses’ experiences: balancing efficiency gains with information safety concerns, role transformation by emerging professional responsibilities, and potential patient benefits with barriers to effective use.

### Comparison With Prior Work

Previous studies have highlighted the practical value of LLMs as clinical assistants, including improving patient education materials and supporting clinical decision-making [20,21]. However, concerns regarding LLM “hallucinations” and inaccurate information remain significant barriers [22-24]. Consistent with prior studies, nurses in our study recognized the potential benefits of LLM-assisted health education while also expressing concerns regarding information reliability and safety. Our findings also further highlighted an important tension between efficiency and safety: while LLMs may reduce repetitive educational work, they also require nurse oversight to support safe and appropriate information delivery [25]. Similar issues have been reported in clinical decision support systems, where the usefulness of digital tools depends not only on the information they provide but also on how they fit into existing clinical workflows and professional practices [26]. These findings suggest that safe integration of LLM into nursing practice depends not only on technological performance but also on clinical workflows for nurse-led verification and correction.

Contrary to concerns that LLMs might lead to overreliance [25], our findings indicate the opposite view: nurses demonstrated heightened awareness of their professional responsibilities, actively seeking to compensate for gaps in their expertise even when LLMs were available as support. LLMs supported professional development but also amplified cognitive demands and accountability, particularly among less experienced staff.

From the nurses’ perspective, LLM use may help patients to access a broader range of health education information and participate more actively in disease-related decision-making. This finding is consistent with previous research by Dehelean et al [27]. However, participants noted that older patients often have limited digital literacy; their reluctance to use LLM is not due to the lack of valuable content but rather insufficient support in effectively using the technology. This aligns with previous studies showing that older adults, retirees, and individuals with lower educational levels are overrepresented among nonusers [10].

Furthermore, although LLMs have been explored for providing emotional support in psychological care and show considerable potential for demonstrating empathy [28], empathy remains a key element of patient care and person-centered practice. Similar concerns have been reported with electronic health records, where documentation demands may affect direct patient communication [29]. This highlights that LLMs should complement rather than replace human interaction in health care settings [30].

Frontline nurses experienced a complex set of tensions when using LLMs for patient health education. The technology enhanced efficiency, consistency, and reach, yet simultaneously increased professional responsibilities, required careful supervision, and exposed specific patient limitations. Beyond documenting general benefits and concerns of LLMs in health care, this study highlights 3 important practice implications: nurses actively assumed a gatekeeping role rather than relinquishing responsibility; less experienced nurses faced disproportionate pressure, suggesting targeted training needs; and patient barriers were driven not only by content limitations but also by insufficient support for technology use.

### Interpretation Through Normalization Process Theory

Our findings can be further interpreted through the lens of the normalization process theory [31], which focuses on how new practices are incorporated into routine clinical work. Normalization process theory was not used to guide data collection or thematic development in this study; rather, it was applied retrospectively as a secondary interpretive framework to contextualize nurses’ experiences with LLM-assisted health education. From the perspective of coherence, nurses considered the value of LLM-assisted health education by weighing its potential to improve efficiency against concerns about information safety. Regarding cognitive participation, nurses described changes in their roles and responsibilities as well as the need for additional knowledge and support. In relation to collective action, nurses highlighted the importance of integrating LLM use into existing education processes while maintaining professional oversight. Reflexive monitoring was reflected in nurses’ reflections on whether this approach was appropriate for different patients and clinical situations.

Our findings suggest that the integration of emerging technologies into clinical practice depends not only on model performance but also on how these technologies align with nurses’ roles and clinical needs. Rather than replacing nursing work, LLMs may change nurses’ roles, with nurses reviewing interaction records, adapting information to clinical contexts, and addressing patient-specific needs. Future implementation efforts should consider workflow-oriented design strategies that support nurse oversight, contextual adaptation, and patient-centered use in clinical settings while future studies should evaluate changes in workflow processes and workload-related outcomes.

### Ethical Implications

Despite recent AI accountability legislation [32], responsibility in clinical LLM implementation remains unclear. Prior research has also shown that although patients have positive expectations of AI, they remain concerned about the accuracy of AI-generated information and who is accountable when errors occur [33]. Traditional liability frameworks hold that clinicians, as the interface between LLM recommendations and patient care, remain the ultimate decision-makers and bear primary responsibility for outcomes [25]. In the context of patient education, this study further extends this perspective by showing that frontline nurses did not abdicate responsibility for education quality; rather, they continued to review, verify, and contextualize LLM-generated content, positioning themselves as the primary supervisors of patient education.

### Limitations

First, results may be impacted by selection and social desirability bias and are subject to the constraints of self-reporting. Participants’ awareness of the research context and prior exposure to LLM-assisted education may have shaped their perceptions and descriptions of their experiences. Second, this study focused only on nurses’ perspectives and did not include patients’ direct experiences or perceptions regarding LLM-assisted health education. Although nurses could provide clinical observations of patient engagement during the workflow, these observations may not fully capture patients’ own experiences and perceptions. Future studies incorporating patients’ own experiences are needed to provide a more comprehensive understanding. Third, although no substantially new insights emerged during later interviews, all participants were recruited from a single hospital. The findings may reflect the specific organizational culture, clinical workflows, and experiences of nurses within this setting, which may limit the transferability of findings to other clinical contexts. Future multicenter mixed methods studies are needed to further explore these findings.

### Conclusions

Even in the era of digital health, the core of nursing health education remains accurate and efficient delivery of information [34]. This study suggests that, from nurses’ perspectives, LLM use may support more efficient information delivery while also introducing new considerations related to information safety and clinical oversight. Rather than replacing nurses’ roles, LLM use was perceived to contribute to changes in nurses’ roles and responsibilities, along with increased competency demands. This study also showed potential patients benefits and changes in nurse-patient communication. Future research should further examine how LLMs can be safely integrated into clinical nursing workflows, considering nurse oversight needs, workflow adaptation, and patient characteristics.

## Supplementary material

10.2196/102846 Multimedia Appendix 1 Model details and semistructured interview guide.

10.2196/102846 Checklist 1 SRQR checklist.

## Abbreviations

LLM: large language model
RCT: randomized controlled trial
SRQR: Standards for Reporting Qualitative Research
SSPEC: site-specific prompt engineering chatbot
