# Supplementary material for: Integrating Large Language Models Into Patient Health Education: Interview Study Among Nurses
Source: J Med Internet Res. 2026 Nov 12;28:e102846. doi: 10.2196/102846 (PMC13759179; doi:10.2196/102846)
Supplement: Supplementary file 2 [file jmir-v28-e102846-s001.docx]

Supplementary Material

**Version used**

All interviewed nurses used the locally deployed hfl/chinese-alpaca-2-7b-based SSPEC platform described.

**System configuration and response generation**

All interviewed nurses used the same locally deployed SSPEC configuration. The study team performed no additional study-specific or HSCT-specific fine-tuning. Retrieval used Erlangshen-SimCSE-110M-Chinese embeddings and a local dense vector index constructed from hospital-approved HSCT protocols, patient-education materials, and national guidelines. Paragraph-level chunks were ranked by cosine similarity, the top three passages were retrieved, and the prompt combined a fixed Simplified Chinese system instruction, the retrieved passages, the current question, and dialogue history.

**Safety measures**

Safeguards included the approved local knowledge base, a retrieval-use indicator, standardized training on hallucination risk, and nurse review. The indicator showed whether retrieved knowledge-base content had been used, and nurses reviewed and corrected outputs in health education.

**Data security and governance:**

Patient questions, locally generated speech-to-text transcripts where voice input was used, retrieved passages, model outputs, and coverage-monitoring state were processed on a hospital workstation. The language model, retrieval index, speech-to-text module, and coverage-monitoring module ran on premises without external model API calls or data transfer. Accordingly, these data were not transmitted to, retained by, or used for training by an external model provider. The parent RCT protocol required study data to be de-identified and personal identifiers to be stored separately under strict security. Direct identifiers were not required for SSPEC question processing. Input-side privacy protection relied on nurse-supervised use and separation of identifiers rather than an automated identifier-blocking filter. If identifying information appeared in a transcript, it remained within the local hospital environment and was removed during de-identification before research use.

**Figure 1 A screenshot of our technology**

**Semi-structured interview guide**

Opening question

1. Could you describe your experience participating in the LLM-assisted health education workflow?

Example probes:

Can you describe a specific situation or experience?

What was your role during this process?

2.How did the LLM-assisted health education workflow influence your clinical practice and role in patient education?

Example probes:

Did your responsibilities change?

Did it affect how you provided education or monitored patient information

3.What changes, benefits, or difficulties did you experience during this workflow?

4. Based on your experience, what improvements or considerations would support future use of LLM-assisted health education?
